# Beclin-1–Dependent Autophagy Improves Outcomes of Pneumonia-Induced Sepsis

**DOI:** 10.3389/fcimb.2021.706637

**Published:** 2021-06-15

**Authors:** Azadeh Nikouee, Matthew Kim, Xiangzhong Ding, Yuxiao Sun, Qun S. Zang

**Affiliations:** ^1^ Burn & Shock Trauma Research Institute, Department of Surgery, Loyola University Chicago Stritch School of Medicine, Maywood, IL, United States; ^2^ Department of Pathology, Loyola University Chicago Stritch School of Medicine, Maywood, IL, United States; ^3^ Department of Surgery, University of Texas Southwestern Medical Center, Dallas, TX, United States

**Keywords:** autophagy, pneumonia, sepsis, infection, inflammation

## Abstract

**Objective:**

We previously demonstrated that promoting Beclin-1–dependent autophagy is cardiac protective during endotoxemia shock, suggesting that autophagy-based approaches may become a promising therapeutic strategy for sepsis. In this study, we applied both genetic and pharmacological approaches to evaluate whether Beclin-1 activation improves sepsis outcomes in a model of pneumonia-induced sepsis.

**Methods:**

Sepsis was induced in mice by *Klebsiella pneumoniae* infection *via* intubation, and outcomes of clinical sickness scores, systemic infection, inflammation, survival, and pulmonary pathology were examined. Evaluation of Beclin-1 activation was achieved by comparing strains of C57BL/6J wild type and *Becn1F121A* that carries a transgenic expression of Beclin-1–active mutant F121A, and by comparing animal groups treated with Beclin-1–activating peptide, Tat-beclin-1 peptide (TB-peptide), or with vehicle control. The status of autophagy in the lung tissue was examined in autophagy reporter mice, CAG-RFP-EGFP-LC3, by fluorescence microscopy.

**Results:**

Pulmonary infection by *K. pneumoniae* produced an insufficient, maladaptive autophagy in the lung. Activation of Beclin-1 by forced expression of active mutant *Becn1F121A* or by treatment with TB-peptide enhanced autophagy and significantly reduced sickness scores, systemic infection, and circulating and pulmonary cytokine production. Both approaches demonstrated notable benefits in limiting post-infection pathogenesis in the lung, such as decreases in alveolar congestion, hemorrhage, infiltration of inflammatory cells, and alveolar wall thickness.

**Conclusion:**

Data suggest that targeted activation of Beclin-1 alleviates adverse outcomes of pneumonia-induced sepsis, and thus, possess a therapeutic potential.

## Introduction

Sepsis is a life-threatening condition of organ dysfunction caused by a deregulated host response to infection (Singer et al., 2016). Despite improvements in antibiotic therapies and critical care techniques (Levy et al.), sepsis remains a leading cause of death in critical care units (Singer et al., 2016), and its reported incidence is still increasing (Iwashyna et al., 2010). Understanding of the pathological mechanisms and exploration of new therapeutic interventions for sepsis are in urgent need.

Research in our laboratory has been using the heart as a model to investigate the pathophysiologic mechanisms of sepsis-induced multi-organ failure. We previously demonstrated that sepsis triggers damage in mitochondria, resulting in an overproduction of mitochondria-derived danger-associated molecular patterns (DAMPs), such as mitochondrial reactive oxygen species (mtROS) and fragmented mitochondrial DNA (mtDNA) (Zang et al., 2007; Zang et al., 2012a; Zang et al., 2012b). These harmful molecules exacerbate myocardial inflammation and cardiac dysfunction during sepsis (Zang et al., 2012b; Yao et al., 2015). We recently examined the impact of autophagy, a survival lysosome-dependent process of removing damaged proteins and organelles (Mizushima and Levine, 2010), on cardiac performance during endotoxemia induced by lipopolysaccharide (LPS), a major pathogen-associated molecular patterns (PAMPs) from gram-negative bacteria (Sun et al., 2018). We discovered that enhancing autophagy *via* the specific activation of Beclin-1, a universally expressed autophagy initiation factor (Liang et al., 1998; Liang et al., 1999), protects mitochondria, reduces mitochondrial DAMPs, and alleviates inflammation in the heart during endotoxemia (Sun et al., 2018). More importantly, the specific activation of Beclin-1, either genetically or pharmacologically, significantly improves cardiac performance under the challenge of septic shock by LPS (Sun et al., 2018). This study leads us to postulate that the targeted activation of autophagy factors may become an effective approach to boost adaptive autophagic responses, and thus, improves outcomes in sepsis.

Clinically, pneumonia-induced sepsis is one of the most common sepsis etiologies and is associated with the highest rate of mortality (Esper et al., 2006; Mayr et al., 2014). To enhance the clinical relevance of our investigation, we established a preclinical mouse model of pneumonia-induced sepsis according to literature, in which sepsis is induced by an intratracheal injection of bacteria (Zang et al., 2012a; Zang et al., 2012b). In the studies summarized in this report, we evaluated the impact of Beclin-1–dependent autophagy on the outcomes after pneumonia-induced sepsis by both genetic and pharmacologic approaches. Our results suggest that autophagy-based approaches provide a promising therapeutic potential for sepsis.

## Materials and Methods

### Experimental Animals

Wild type C57BL/6 mice were obtained from Charles River laboratories (Massachusetts, MA) and in-campus mouse breeding core facility at The University of Texas Southwestern Medical Center (UTSW). All animals were conditioned in-house for 5 to 6 days after arrival with commercial diet and tap water available at will. Mouse strains carrying autophagy reporter CAG-RFP-EGFP-LC3 (Li et al., 2014), a F121A mutation in *beclin-1* (*Becn1* ^F121A/F121A^) (Fernandez et al., 2018), and haploinsufficient for *beclin-1* (*Becn1* ^+/−^) (Qu et al., 2003) were previously developed. Animal work described in this study was reviewed and conducted under the oversight of UTSW Institutional Animal Care and Use Committee and conformed to the “Guide for the Care and Use of Laboratory Animals” when establishing animal research standards.

### Pneumonia-Related Sepsis Model

(1) Preparation of inoculum: Upon received, *Klebsiella pneumoniae* type 3 (ATCC, Rockville, MD, catalog number 43816) was inoculated into 2 ml nutrient broth medium (NBM) (Difco nutrient broth, BD Diagnostic, Burlington NC; supplier number 23400) and amplified on shaker incubator at 37°C for overnight. 0.5 ml of this bacterial suspension was further cultured in 50 ml NBM at 37°C for about 3.5 h, growing to log phase till the OD_600_ density reached 0.8 when measured by spectrophotometer. The culture was then aliquoted and stored at 80°C until used. To prepare viable bacteria used for infection, the frozen bacteria stock was thawed, inoculated, and cultured to log phase as described above. This bacterial preparation was kept at 4°C and was ready to infect animals within the next three days. To determine the colony-formation units (CFU), a small amount of this bacterial suspension was diluted by folds of 10^7^ and 10^8^, and multiple 100-μl diluents were plated on 10- cm nutrient agar plates (made with Difco nutrient agar from BD Diagnostic, Burlington NC; supplier number 21300). After incubation at 37°C for overnight, bacterial colonies were counted and the number of CFU was calculated. (2) Induction of sepsis: Sepsis was induced by endotracheal delivery of *K. pneumoniae via* intubation by otoscope. The dose of bacteria inoculated into the animals was 3 × 10^7^ CFU per 25 grams body weight. The prepared bacterial suspension was adjusted to concentration at 3 × 10^7^ CFU per 50 μl using NBM. 10- to 12-week-old male mice were weighed individually to determine the exact amount of bacterial suspension injected into each animal. Mice were anesthetized with 90 mg/kg ketamine/10 mg/kg xylazine cocktail *via* i.p. prior to intubation. Bacterial suspension, 3 × 10^7^ CFU per 50 μl per 25 g body weight, was used for infection and the uninfected group received 50 μl PBS as control. Tat-Beclin-1 peptide (TB-peptide) was synthesized according to published sequence (Pietrocola et al., 2016) by NonoPep (Shanghai, China). In the case that when animals receiving TB-peptide treatment, it was administered i.p. at a dose of 16 mg/kg in 100μl PBS post inoculation.

### Post-Infection Monitoring and Evaluation of Sickness Conditions

Following infection, animals were monitored three times daily for a period of 5-day post infection. For each animal, the progress of clinical sickness was recorded according to a pre-designed scoring system that evaluates the parameters of overall physiological conditions, appearance, movements, behavior, respiration, and other abnormalities (**Table 1**). The assessment allowed the determination of humane endpoints in survival studies, in which a total score above 6 or a single category score above 3 indicates fatality. In addition, this rating system allowed the comparison of an overall progress in sickness between groups with or without a genetic trait or a treatment.

**Table 1:** Criteria of Clinical Sickness Scores.

		Normal	Mild	Moderate	Severe
	Score	0	1	2	3
Body Weight		0-5% decrease	6-10% decrease	11-15% decrease	>20 % decrease
Body Condition Score		good muscle mass & body fat	slight loss of muscle mass & body fat	moderate loss of muscle mass & body fat	pelvic bones, ribs, and/or spine visible
Appearance	Posture	Balanced & symmetrical	Slight asymmetry	Moderate difference from cohorts	hunched or asymmetrical
	Coat	well groomed with sheen	loss of sheen & slightly ruffled; mild dermatitis	dull, rough appearance; moderate dermatitis	ungroomed/unkempt, and thin; persistent, nonhealing dermatitis >20% body area
	Eyes	Bright, clear, no discharge	slightly closed / no discharge	slightly closed with discharge and/or swelling	closed with discharge or open with swelling
Movement		moving well with no impediment	moving slowly or with slight difficulty / irregularity	moving slowly with difficulty or restlessness	not moving; staying away from cohorts; obsessive activity; not able to obtain food and/or water normally
Natural (Unprovoked) Behavior		interacts with cohorts & environment	mild interest in environment & minimal interaction with cohorts	isolated from cohorts & disinterested in environment	vocalizing or unaware of surroundings, Immobile or hyper reactive; self-mutilation
Respiration		normal (163-220 breaths/min.)	mild increase or decrease (difference <20 %)	moderate increase, or labored (+/- 25%)	rapid or severely labored (+/-50%)
Other Signs		no other issues	mild local issue e.g. scratch or licking a part of its body frequently	moderate systemic issue (e.g. slightly swollen abdomen)	serious systemic issue (e.g. prolapsed organ; rectal prolapse; bleeding)

### Evaluation of Systemic Infection

When animals were sacrificed, blood was collected using Vacutainer rapid serum tubes (RST) (BD Diagnostics, Franklin Lakes, NJ; catalog number 368774). Organs were harvested and homogenized in PBS. The presence of bacterial infection was examined by culturing the blood or tissue lysates on nutrient agar plates for overnight at 37°C. Numbers of colony formation were normalized with the volume of blood or with the amount of protein in tissue lysates.

### Histology Analysis of Lung Injury

Fresh lung tissues were perfused in PBS, followed by fixation in 4% paraformaldehyde, and then left in the fixation buffer for 24 h at 4°C. For dehydration, fixed tissues were first transferred to 10% sucrose/PBS for 24 h, then to 18% sucrose/PBS for another 24 h, and both steps were performed at 4°C. Tissue samples were embedded in OCT, sectioned at 8 μm, air-dried, and stored at −80°C until used. Frozen slides were thawed, rehydrated, and subjected to histological staining. Lung injury were quantified by an investigator blinded to the treatment groups as described previously (Jiang et al., 2017). In brief, the following four pathological changes were measured and normalized by the total areas examined: alveolar congestion, hemorrhage, infiltration of inflammatory cells or aggregation of neutrophils in air space or the vessel wall, and alveolar wall thickness. Twenty random high-power fields were examined per animal.

### Detection of Autophagy by Fluorescence Microscopy

Autophagy in the lung tissue was evaluated using the mouse strain of CAG-RFP-EGFP-LC3 (Li et al., 2014). OCT-embedded tissue slides were sealed with DAPI/antifade mounting solution (Thermo Fisher Scientific, Rockford, IL; catalog number 36931) and examined under Zeiss Axiovert 200M inverted fluorescence microscope at 20× magnification.

### Preparation of Serum and Tissue Lysates and Cellular Fractions

Freshly collected blood was immediately centrifuged at 3,000*g* for 15 min at 4°C to isolate serum. The serum preparations were then allocated and stored at −80°C until analyzed. Tissues were harvested, washed in PBS, snap clamp frozen, and kept at −80°C. Tissue lysates were prepared using tissue protein extraction reagent (Thermo Fisher Scientific, Rockford, IL; catalog number 78510). Protein concentrations were quantified using detergent compatible Bradford assay kit (Thermo Fisher Scientific, Rockford, IL; catalog number 23246).

### Measurements of Cytokines by Enzyme-Linked Immunosorbent Assay (ELISA)

Cytokine levels in serum or in total tissue lysates were measured using Bio-Plex Mouse Cytokine Panel A 6-Plex (Bio-Rad, Hercules, CA; catalog number M6000007NY) according to vendor’s instructions. Results were normalized by volume of serum samples or by protein amount in tissue lysates.

### Statistical Analysis

Results were expressed as mean ± SEM using the indicated number of experiments or mice. Student t-tests were applied for comparisons between groups. Nonparametric Kruskal-Wallis H test was applied to compare the mean rank of multiple pain score groups. Kaplan-Meier survival curves and relevant Log-rank statistical test were applied in the survival study. Differences were considered statistically significant when *p* ≤ 0.05, and all samples were tested at least in triplicate.

## Results

### Beclin-1 Activation Boosts Autophagy Response in the Lung Under Septic Infection

In the pneumonia-induced sepsis model, mice were infected with gram-negative *K. pneumoniae via* intubation. Infection dose at 3 × 10^7^ CFU per mouse resulted in a mortality rate about 60% to 70% during the 5-day post-infection period, and bacteremia was confirmed 24 h post infection. Based on published results as well as observations in our laboratory, male and female mice showed significantly different susceptibility to respiratory and systemic symptoms in the pneumonia-induced sepsis model (Kadioglu et al., 2011). Thus, male but not female mice were chosen for the experiments presented in this report.

Autophagy reporter mice, CAG-RFP-EGFP-LC3, were chosen to examine autophagy flux in the lung tissue of the pneumonia-induced sepsis model. In these mice, the expression of a tandem red fluorescent protein (RFP)-EGFP-LC3 fusion protein was constructed under the CAG promoter (Li et al., 2014). Taking the advantage of differences in acid sensitivity between RFP and EGFP, both EGFP and RFP fluorescence are present during autophagosomal maturation (pH 5.9), indicating the early step of autophagic flux, whereases RFP signals in acid autolysosomes (pH 4.5) which formation occurs in a later step.

**Figure 1:**
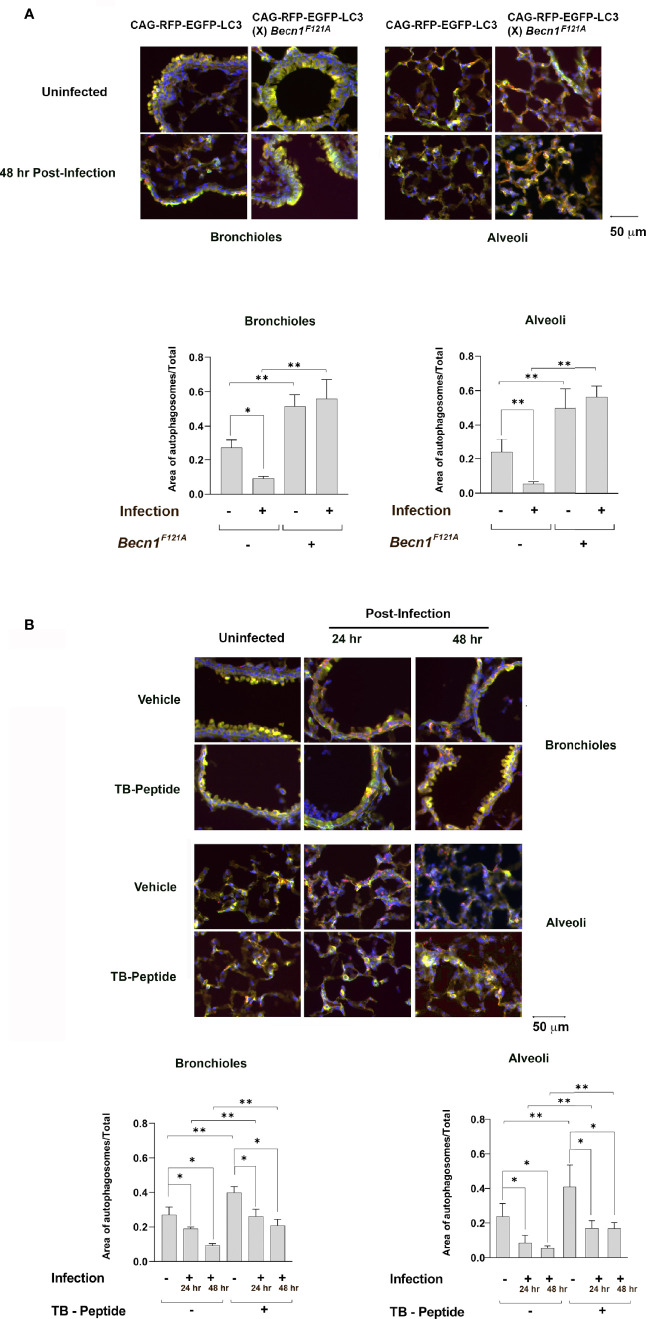
Lung autophagy in response to pneumonia-induced sepsis and the effects of Beclin-1 activation. **(A)** Autophagy reporter CAG-RFP-EGFP-LC3 mice or mice of CAG-RFP-EGFP-LC3 (X) *Becn1* ^F121A/F121A^ were given septic infection of *K. pneumoniae* (3X10^7^ CFU/mouse) or PBS (uninfected) *via* intubation. Lung tissue samples were harvested 48 h post infection, and lung tissue slides were evaluated under fluorescence microscopy. Neutral autophagosomes, shown in yellow, allow emissions from both EGFP (green) and RFP (red). Acid autolysosomes, shown in red, are due to signals from RPF. DAPI in blue indicates nucleic acid staining. **(B)** CAG-RFP-EGFP-LC3 mice receiving *K. pneumoniae* infection or PBS (uninfected) were given TB-peptide (16.5 mg/kg, i.p.) or PBS control vehicle 1 h post-infection. Lung tissue samples were harvested 24 and 48 h post infection, and lung tissue slides were evaluated under fluorescence microscope as described in **(A)** In both **(A, B)** autophagosome areas were quantified as ratios to the total area examined. Images are representative of n ≥ 5 animals per group and ten random high-power fields were examined per animal. All values are means ± SEM. Significant differences are shown as * for uninfected *vs*. infected, ** for WT *vs*. *Becn1* ^F121A/F121A^ **(A)** or for TB-peptide treated *vs*. untreated **(B)** (*p* < 0.05, student t-test).

A mouse strain with transgenic expression of active mutant F121A in *beclin-1*, *Becn1* ^F121A/F121A^, was utilized as a genetic approach to up-regulate autophagy (Fernandez et al., 2018). We generated a new strain by crossing *Becn1* ^F121A/F121A^ with CAG-RFP-EGFP-LC3, and its autophagy response in the lung under *K. pneumoniae* infection was compared with that in CAG-RFP-EGFP-LC3 mice. As shown in **Figure 1A**, mice were infected with *K. pneumoniae* and PBS was used in the uninfected group. In the lung tissue samples harvested 48 h post infection, the status of autophagy in areas of alveoli and bronchioles were evaluated under fluorescence microscopy. Presence of autophagosomes was shown in yellow, due to overlying the colors of green from EGFP emission and red from RFP. Autolysosomes were shown in red, due to signals from RPF. DAPI for nucleic acid staining was used to visualize the location of cells. In CAG-RFP-EGFP-LC3 mice, an infection-associated reduction was evident in the autophagosome population, suggesting an inhibition in initiating autophagy flux. As expected, forced expression of active mutant of Beclin-1 resulted in a significantly enhanced signal of autophagosomes in CAG-RFP-EGFP-LC3 (X) *Becn1* ^F121A/F121A^ mice, in consistent with the function of Beclin-1 as an autophagy initiation factor.

Previous research from others and ours showed that a cell-permeable Beclin-1 activating peptide, Tat-beclin-1 (TB-peptide), is a pharmacological approach of promoting autophagy *in vitro* and *in vivo* (Shoji-Kawata et al., 2013; Pietrocola et al., 2016; Shirakabe et al., 2016; Sun et al., 2019; Sun et al., 2021). We evaluated its effects in the model of pneumonia-induced sepsis, choosing a dose that inducing sufficient autophagy without causing detectable toxicity (Sun et al., 2019; Sun et al., 2021). As shown in **Figure 1B**, CAG-RFP-EGFP-LC3 mice were given bacterial infection and received the treatment of TB-peptide (16 mg/kg, i.p.) 1 h post-infection. PBS was given in the vehicle control groups. Lung tissue samples harvested 24 and 48 h post infection were evaluated under fluorescence microscopy. In the vehicle-treated groups, the infection caused a visible decrease in the population of autophagosomes in the areas of both alveoli and bronchioles, suggesting an inhibitory effect on starting autophagy. However, the treatment of TB-peptide boosted autophagy, as demonstrated by the intensified signals of autophagosomes in the areas of alveoli and bronchioles. These results confirmed that TB-peptide promotes autophagy in the lung under the challenge of septic infection.

### Beclin-1–Dependent Autophagy Controls Local and Systemic Infection and Reduced Sickness Scores

**Figure 2:**
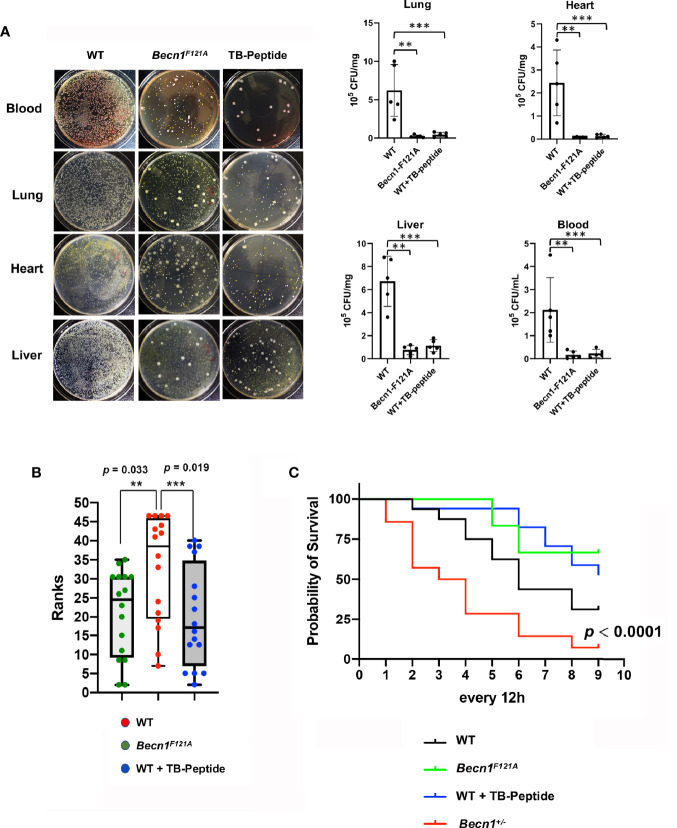
Effects of Beclin-1–dependent autophagy on local and systemic infection, clinical pain scores, and survival in pneumonia-induced sepsis. Mice of WT, *Becn1* ^F121A/F121A^, or *Becn1* ^+/−^ were given septic infection by *K. pneumoniae* (3X10^7^ CFU/mouse, intubation). A parallel group of infected WT mice were treated with TB-peptide (16.5 mg/kg, i.p.) 1 h post-infection. **(A)** To evaluate infection, blood was collected, and organs harvested 48 h post infection. Blood serum and tissue lysates were plated on nutrient agar plates and incubated at 37°C for overnight. Bacterial colony formation was normalized with serum volume or lysate protein concentration. All values are means ± SEM. Significant differences are shown as ** for WT *vs*. *Becn1* ^F121A/F121A^, and *** for TB-peptide treated *vs*. untreated (*p* < 0.05, n = 5, student t-test). **(B)** Post-infection physiological conditions were monitored and scored according to **Table 1** at indicated time points. Kruskal –Willis H test for mean rank adjusted for multiple testing was applied, and differences were considered statistically significant when *p* ≤ 0.05 (** for WT *vs*. *Becn1* ^F121A/F121A^ and *** for TB-peptide treated *vs*. untreated). **(C)** Survival curves were compared by a log-rank test, and differences were considered statistically significant when *p* ≤ 0.05. In B and C: n = 16 for WT, n = 12 for *Becn1* ^F121A/F121A^, n = 17 for WT plus TB-peptide, and n = 14 for *Becn1* ^+/−^.

To examine the impacts of Beclin-1–dependent autophagy on sepsis outcome after pneumonia infection, mice of wild type (WT) and *Becn1* ^F121A/F121A^ were given *K. pneumoniae* infection. In parallel experimental groups, WT mice received the treatment of TB-peptide. Degrees of local infection in the lung tissue and systemic infection in blood and in distant organs, such as heart and liver, were compared. As shown in **Figure 2A**, examined at 48 h post infection, a 10-fold or more decreases in colony formation were observed in mice with Beclin-1 activation, either *Becn1* ^F121A/F121A^ mice or TB peptide–treated mice, when compared with that in the WT counterparts.

Additional parallel groups of mice were monitored for a period of 5-day post infection, clinical sickness scores were evaluated by observing the body physiological conditions, appearance, movements, behavior, respiration, and other abnormalities, as described in the method section (**Table 1**). Statistical analysis was applied according to published methods (La Colla et al., 2009), and data revealed that activation of Beclin-1 either genetically (*Becn1* ^F121A/F121A^) or pharmacologically (TB-peptide) significantly reduced the overall sickness scores in response to pneumonia-induced sepsis (**Figure 2B**).

Lastly, survival rates of infected mice of WT, *Becn1* ^F121A/F121A^, and WT plus TB-peptide treatment were examined (**Figure 2C**). No statistical significance was detected when *Becn1* ^F121A/F121A^ mice or TB peptide–treated mice were compared with WT or vehicle-treated mice under the indicated experimental setting. However, a strain of haploinsufficient of Beclin-1 (*Becn1* ^+/−^) showed a significantly decreased survival rate when compared with the rest of the groups. The result suggests that Beclin-1 signal is at least essential for survival under the challenge of pneumonia-induced sepsis.

### Beclin-1–Dependent Autophagy Attenuates Pulmonary and Systemic Cytokine Productions in Pneumonia-Induced Sepsis

**Figure 3:**
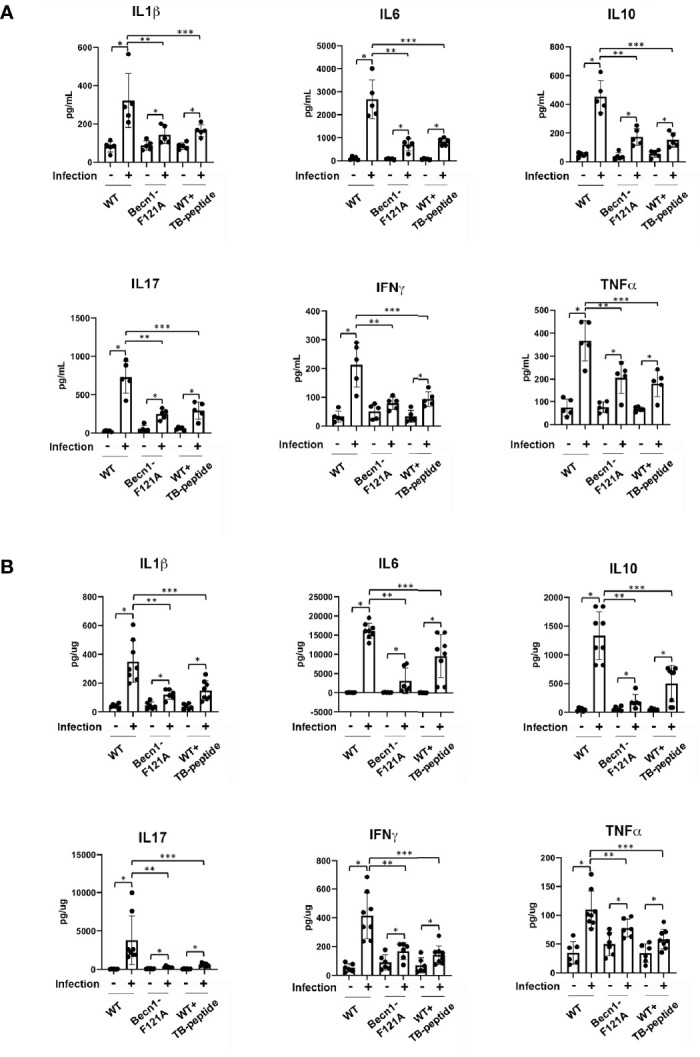
Beclin-1–dependent autophagy attenuates systemic cytokines and pulmonary cytokines in pneumonia-induced sepsis. Mice of WT and *Becn1* ^F121A/F121A^ were given septic infection by *K. pneumoniae* (3X10^7^ CFU/mouse, intubation) or PBS (uninfected). In parallel groups, infected and uninfected mice were treated with TB-peptide (16.5 mg/kg) or PBS vehicle 1 h post-infection. Serum was collected and lung tissue harvested 48 h post-infection. Cytokines in serum **(A)** and in lung tissue lysates **(B)** were measured by ELISA assays. All values are means ± SEM. Significant differences are shown as * for uninfected *vs*. infected, ** for WT *vs*. *Becn1* ^F121A/F121A^, and *** for TB-peptide treated vs. untreated (*p* < 0.05, n ≥ 5, student t-test).

Whether increasing Beclin-1–dependent autophagy has an effect on the control of overwhelming inflammation induced by pneumonia-induced sepsis was also addressed. Mice of WT or *Becn1* ^F121A/F121A^ were given septic infection by *K. pneumoniae*. In parallel groups of WT mice, TB-peptide or control vehicle PBS was administered 1 h post infection. At 48 h post-infection, blood was collected and lung tissue harvest. Cytokines present in serum and in the lung tissue lysates were compared. As shown in **Figures 3A, B**, activation of Beclin-1 by forced expression of *Becn1* ^F121A/F121A^ or by TB-peptide provided similar levels of substantial reduction in circulating cytokines, as well as in pulmonary cytokines, indicating that Beclin-1–dependent autophagy provides anti-inflammatory effects during sepsis.

### Beclin-1–Dependent Autophagy Alleviates Pulmonary Pathology in Pneumonia-Induced Sepsis

**Figure 4:**
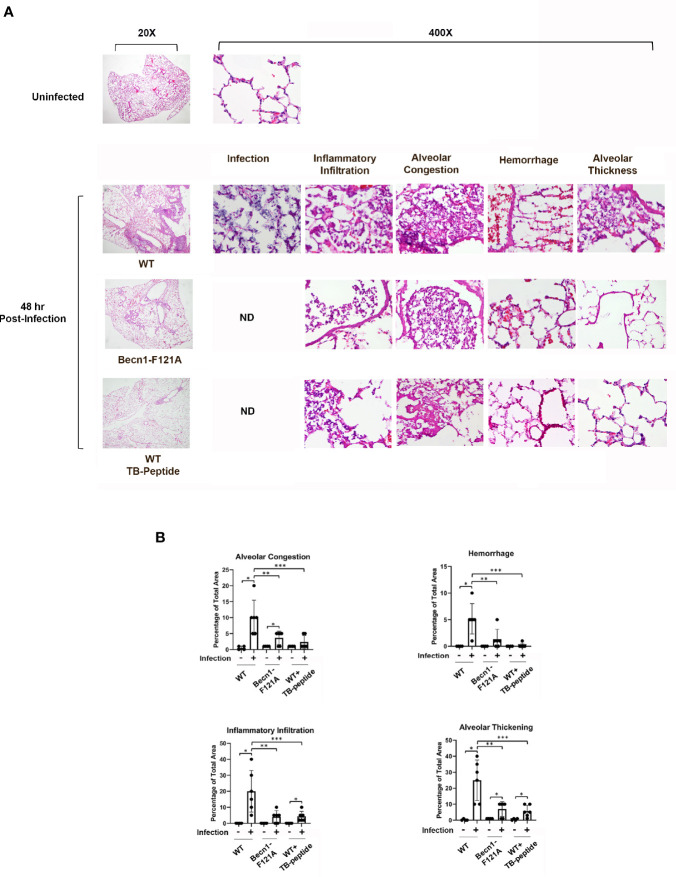
Beclin-1–dependent autophagy limits the progress of pulmonary pathology in pneumonia-induced sepsis. Mice of WT and *Becn1* ^F121A/F121A^ were given septic infection by *K. pneumoniae* (3X10^7^ CFU/mouse, intubation) or PBS in the uninfected group. In parallel groups, infected and uninfected WT mice were treated with TB-peptide (16.5 mg/kg, i.p.) or PBS vehicle 1 h post-infection. Lung tissue was harvested 48 h post-infection, and tissue slides were stained with Hematoxylin and Eosin (H&E). **(A)** H & E images of lung histology were analyzed under Olympus BX43 microscope at magnifications of 20X and 400X. Images are representative of n=5 per group. **(B)** Areas of inflammatory infiltration, alveolar congestion, alveolar thickness, and hemorrhage were quantified as percentage of total area using Image J All values are means ± SEM. Significant differences are shown as * for uninfected vs. infected, ** for WT *vs*. *Becn1* ^F121A/F121A^, and *** for TB-peptide treated *vs*. untreated (*p* < 0.05, n = 5, student t-test).

Since infection was induced in the lung in this sepsis model, pulmonary pathology was examined according to criteria described in literature (Jiang et al., 2017). Degrees of alveolar congestion, hemorrhage, infiltration of inflammatory cells or aggregation of neutrophils in air space or the vessel wall, and alveolar wall thickness were compared between infected mice of WT, *Becn1* ^F121A/F121A^, and WT receiving TB-peptide treatment. As shown in **Figure 4A**, the lung tissue slides were subjected to Hematoxylin and Eosin (H&E) staining. At 48 h post infection, areas of bacterial infection, infiltration of immune cells, hemorrhage, alveolar congestion, and increases in alveolar wall thickness were dramatic in the lung of WT mice, in contrast to those in mice of *Becn1* ^F121A/F121A^ and WT treated with TB-peptide. Especially, presence of bacterial infection was barely detected in the latter two groups, suggesting a stronger bactericidal activity in response to Beclin-1 activation. Quantification of those pathological areas in percentage of the total areas examined showed that activation of Beclin-1, either by mutation at F121A or by treatment with TB-peptide, significantly reduced lung injury in response to the septic challenge (**Figure 4B**).

## Discussion

We previously showed that activation of autophagy initiator Beclin-1 provided a benefit of cardiac protection during endotoxemia (Sun et al., 2018). In this investigation, we designed a study to further address whether targeted activation of Beclin-1 possesses therapeutic potential for sepsis using a previously established model of pneumonia-induced sepsis, which has more clinical relevance (Zang et al., 2012a; Zang et al., 2012b). In this model, pulmonary infection of gram-negative *K. pneumoniae* was introduced *via* intubation, and sepsis occurrence was confirmed by systemic infection. The impact of Beclin-1 activation was addressed using both genetic and pharmacological approaches. In our experimental setting, we found that promoting Beclin-1–dependent autophagy significantly improved sepsis outcomes, including reductions in sickness scores, infection, and inflammation. Examination of lung pathology revealed that enhanced Beclin-1 signaling alleviated lung injury, as shown by limiting the degrees of alveolar congestion, hemorrhage, infiltration of inflammatory cells, and alveolar wall thickness, and therefore, alleviated lung injury. Together, the data provide a strong support for the notion that Beclin-1–dependent autophagy presents therapeutic values to improve sepsis outcomes.

In this study, we used a strain of autophagy reporter mice CAG-RFP-EGFP-LC3 to examine the status of lung autophagy post septic infection. We found that infection hindered autophagy flux at the time points examined, suggesting an insufficient, maladaptive autophagy response (**Figure 1** **)**. A previous study using a mouse model of polymicrobial sepsis, cecal ligation and puncture (CLP), showed elevated autophagy in the lung tissue (Lee et al., 2014). This discrepancy is likely due to the differences in the time points chosen for experiments and/or the septic responses in different models. Autophagy is triggered as a defense mechanism during the early stage of sepsis but attenuated with the progression in severity. For example, signals detected in the heart of a mouse model of endotoxemia and in the liver of CLP sepsis indicate that a decline in autophagy is tightly associated with the occurrence of organ failure (Chien et al., 2011; Takahashi et al., 2013; Sun et al., 2018). In the study reported here, the lung tissue was evaluated at 48 h post infection, at which time point signs of obstructed lung function such as rapid and labored respiration were observed. Nonetheless, we confirmed that activation of Beclin-1 either genetically by a forced expression of activation mutant *Becn1F121A* or pharmacologically by an activating peptide, TB-peptide, indeed dramatically enhanced autophagy signaling in the lung under the condition of infection.

Bacterial clearance by macrophages *via* phagocytosis is the first step of defense to remove invaded pathogens. Enhanced bactericidal activity of macrophages was previously observed in *Becn1F121A* mice, which carry an active mutant Beclin-1 (Fernandez et al., 2018). The anti-pathogenic function of autophagy in macrophage defense capacity was also suggested in models with reduced expression of autophagy factors. For example, atg7 deficiency led to impaired host defense in macrophages, and thus resulting in magnified infection, inflammation, and worsened injuries in the lung of animals infected by *K. pneumoniae* (Ye et al., 2014). Consistent with these studies, we observed that activate mutant Beclin-1 *Becn1F121A* or treatment with TB-peptide provided a significant reduction in infection, locally in the lung and systemically in blood and in distant organs, in response to infection by *K. pneumoniae* (**Figures 2** and **4**). In this report, infection was examined at the tissue level rather than in cell types such as in macrophages. In our ongoing investigations, we indeed observed that TB-peptide improved bactericidal activities in cultured macrophages (data not shown). However, roles of other cell types in this process of pathogen clearance may not be excluded since changes in autophagy alter metabolic and inflammatory responses in immune cells as well as non-immune cells of the host body (Painter et al., 2020). Crosstalk between different cell types plays an important role in maintaining the microenvironment that is critical for pathogen survival. Further investigations regarding how host autophagy may be utilized to eradicate evaded microbial pathogens will reveal new opportunities for developing effective therapies for sepsis.

We detected that promoting Beclin-1–dependent autophagy has an anti-inflammatory effect in this model of pneumonia-induced sepsis, as shown by quantification of cytokine production (**Figure 3**) and the evaluation of lung injury (**Figure 4**). The results are consistent with previous observations in models of endotoxemia (Sun et al., 2018) and CLP (Lee et al., 2014). Autophagy interacts with inflammation at multiple layers, and one of those pivotal component mediators are ROS. It is well known that sepsis and acute injuries trigger a surge in ROS production, leading to oxidative stress that contributes to the induction of overwhelming inflammation (Zang et al., 2007; Zang et al., 2012b). Though intracellular ROS are generated at multiple locations, ROS from mitochondria constitute a main portion. In mitochondria, mtROS are produced as by-products of respiratory chain reaction. Upon challenge under pathological conditions, functional deficient and/or structural disrupted mitochondria release various harmful molecules including mtROS that function as DAMPs to stimulate inflammation. Autophagy, on the other hand, can remove dysfunctional organelles, such as mitochondria, through its “self-eating” process, and thus, to control the production of mitochondria-derived DAMPs. In mice subjected to endotoxemia, mitochondrial damage induces inflammation *via* activation of NLRP3-dependent inflammasome in macrophages (Nakahira et al., 2011). In the same model, stimulating Beclin-1–dependent autophagy improves the quality control of mitochondria and reduces inflammation in the heart (Sun et al., 2018), suggesting a mechanism of using autophagy as an approach to mitigate inflammation. We expect similar events occurred in the lung during pneumonia-induced sepsis, and detailed molecular signals in various cell types of the lung tissue will be further interrogated in future studies. It is also noteworthy that the role of autophagy in pathogenesis varies according to different types of conditions, as deleterious side of autophagy was reported in chronic problems such as pulmonary hypertension (Teng et al., 2012) and cardiac hypertrophy (Zhu et al., 2007).

Developing strategies that harness autophagy as effective therapies has received substantial attention in recent years. However, most available autophagy-based approaches have often focused on reagents with broad-spectrum impacts but with limited specificity to autophagy factors (Laplante and Sabatini, 2012). Thus, autophagy-inducing approaches with target specificity and fewer toxic side effects are still limited. A milestone development in this area was the development of TB peptide, which specifically activates Beclin-1. This peptide has been shown to provide beneficial effects in several preclinical disease models (Shoji-Kawata et al., 2013; Pietrocola et al., 2016; Shirakabe et al., 2016). Our evaluation of TB-peptide previously in the endotoxemia model (Sun et al., 2018) and currently in the pneumonia-induced sepsis model have provided novel evidence supporting the notion that pharmacological approaches targeting Beclin-1 signaling possess important therapeutic values for sepsis. In the studies described in this report, our data showed that TB-peptide provided evident benefits in controlling infection and inflammation (**Figures 2** and **4**). It remains unclear whether this treatment can improve survival since no statistical significance was detected (**Figure 2**). Possible reasons behind this observation could be that the sample size is not large enough to reach statistical significance, or the effect of this treatment may only slow down the progress of sepsis symptoms, and therefore, potential benefits in a longer post-infection time course remains to be determined. Nonetheless, the results suggest that TB-peptide, used by itself or in combination with other therapies, has a potential to improve outcomes after sepsis. Additional evaluation of this treatment in other sepsis models or acute injury models will help to fully validate its therapeutic effectiveness and to understand its mechanisms of action.

## Data Availability Statement

The original contributions presented in the study are included in the article/supplementary material. Further inquiries can be directed to the corresponding author.

## Ethics Statement

This study has been reviewed and conducted under the oversight of UTSW Institutional Animal Care and Use Committee and conformed to the “Guide for the Care and Use of Laboratory Animals” when establishing animal research standards.

## Author Contributions

QZ conceived the project, designed the study, and wrote the manuscript. AN, MK, XD, and YS conducted the experiments and contributed to the data analysis. All authors contributed to the article and approved the submitted version.

## Funding

This work is supported by National Institute of Health grant 2R01GM111295-01 to QSZ.

## Conflict of Interest

The authors declare that the research was conducted in the absence of any commercial or financial relationships that could be construed as a potential conflict of interest.
